# PLSCR1/IP3R1/Ca^2+^ axis contributes to differentiation of primary AML cells induced by wogonoside

**DOI:** 10.1038/cddis.2017.175

**Published:** 2017-05-11

**Authors:** Hui Li, Jingyan Xu, Yuxin Zhou, Xiao Liu, L e Shen, Y u Zhu, Zhiyu Li, Xiaotang Wang, Qinglong Guo, Hui Hui

**Affiliations:** 1 State Key Laboratory of Natural Medicines, Jiangsu Key Laboratory of Carcinogenesis and Intervention, Key Laboratory of Drug Quality Control and Pharmacovigilance, China Pharmaceutical University, Nanjing, People’s Republic of China; 2 Department of Hematology, The Affiliated DrumTower Hospital of Nanjing University Medical School, Nanjing, People’s Republic of China; 3 Department of Hematology, The First Affiliated Hospital of Nanjing Medical University, Jiangsu Province Hospital, Nanjing, People’s Republic of China; 4 Department of Chemistry and Biochemistry, Florida International University, Miami, FL, USA

## Abstract

Multiple lines of evidence have demonstrated that increased expression of phospholipid scramblase 1 (PLSCR1) is involved in the differentiation of acute myeloid leukemia (AML) cells by several differentiation-inducing agents including ATRA and phorbol 12-myristate 13-acetate. However, none of these agents can achieve nonhomogenous subcellular distribution of PLSCR1. We have demonstrated that wogonoside possesses differentiation and anti-leukemic effects in AML cell lines by promoting PLSCR1 trafficking into nucleus. Here we report that wogonoside promotes the expression of PLSCR1 and enhances its nuclear translocation and binding to the 1, 4, 5-trisphosphate receptor 1 (*IP3R1*) promoter in AML patient-derived primary cells. Wogonoside activates IP3R1, in turn, promotes release of Ca^2+^ from endoplasmic reticulum, and eventually leads to cell differentiation. Our *in vivo* study further confirms that wogonoside can promote PLSCR1 and IP3R1 expression in primary AML cells and reduce the AML cell counts in engrafted nonobese diabetic/severe combined immunodeficient mice. Taken together, our findings provide new insight into the mechanism of wogonoside-induced differentiation and anti-leukemic effect on primary AML cells, suggesting the therapeutic potential of wogonoside for AML, especially for non-APL AML.

Mutations of hematopoietic genes in progenitors result in acquisition of leukemia conferring deregulated proliferation, impaired differentiation and advantageous survival.^1^ Acute myeloid leukemia (AML) represents a group of malignant clonal disorders of immature myeloid cells where differentiation is inhibited, resulting in accumulation of myeloblasts from different stages and reduced production of normal hematopoietic components.^2^ AML is associated with high morbidity and mortality.^3^ Although complete remission in patients with acute promyelocytic leukemia (APL) has been achieved using targeted therapies (ATRA and/or arsenic trioxide),^4^ the response of non-APL AML patients to the treatment remains poor.^5^ Increasing lines of evidence have demonstrated that several naturally occurring flavonoids have anti-leukemic properties and may serve as potential candidates for leukemia treatment.^6, 7^

Wogonoside, a flavonoid extracted from *Scutellaria baicalensis Georgi* (huangqin), is a metabolite of wogonin with antitumor effect,^8^ and considered as a natural, slow-release prodrug of wogonin.^9^ Our previous studies have demonstrated the anti-leukemic properties of wogonoside, both *in vivo* and *in vitro*, and highlighted the importance of phospholipid scramblase 1 (PLSCR1) in wogonoside-induced differentiation of AML cell lines.^7^ However, the mechanism underlying wogonoside-induced differentiation of AML cells remains poorly understood to date and is not authenticated in primary patient-derived AML cells. Primary AML cells maintain the basic nature and biological activity of AML samples and are more close to clinical practice compare with AML cell lines. These cells exhibit several similarities in terms of morphological structure and functional activity with the organism, and therefore provide a good experimental subject for screening anti-leukemia drugs.

PLSCR1 is a calcium-binding endofacial plasma membrane protein originally shown to accelerate redistribution of plasma membrane phospholipids (PLs) between the inner and outer leaflets following elevation of intracellular Ca^2+^ level.^10^ PLSCR1 also acts as a signaling molecule critical to cell signaling, maturation and apoptosis.^11, 12^ An earlier investigation on protein–protein interaction networks demonstrated that PLSCR1 binds and modulates the activities of several proteins involved in growth factor and cytokine signaling, including epidermal growth factor receptor, c-Src, shc, onzin and the proto-oncogene c-Abl.^13, 14, 15^ Moreover, the observed induction of PLSCR1 by interferons suggests the possible roles of PLSCR1 in immune/stress responses, cell cycle regulation and apoptosis.^16, 17^ Overexpression of PLSCR1 is proposed to inhibit tumorigenesis, induce apoptosis and facilitate the differentiation of myeloid cells.^18, 19^ Recently, potential roles of PLSCR1 in hematopoiesis and leukemogenesis were reported.^11^ It is also demonstrated that PLSCR1 contributes to leukemia cell differentiation induced by ATRA and/or phorbol 12-myristate 13-acetate (PMA). Conversely, silencing PLSCR1 inhibits ATRA/PMA-induced leukemic cell differentiation.^20^ In addition, hematopoietic myeloid precursor cells from *PLSCR1*^-/-^ mice showed defective colony formation and impaired granulocyte terminal differentiation upon stimulation with selective growth factors.^19^ Further investigation revealed that there is significantly decreased expression of PLSCR1 in AML-M1, -M5a and -M5b compared with that in normal bone marrow (BM) cells, and that higher PLSCR1 mRNA levels are associated with significantly longer overall survival in patients with AML.^21^ These findings clearly indicate that PLSCR1 contributes to leukemic cell differentiation and the longer survival of AML patients.

Nuclear PLSCR1 could regulate specific protein function via binding directly to some genes.^22^ Previous research has revealed that PLSCR1 is transported to the nucleus after exposure to cytokine stimulation, where it enhances expression of the inositol 1, 4, 5-triphosphate receptor type 1 (*IP3R1*) gene by directly binding to the promoter region of *IP3R1*.^23^ These data support a mechanism of receptor-mediated nuclear import of PLSCR1 and suggest a potential nuclear function for this plasma membrane protein, which has been a focus of recent research. Inspired by these findings, we investigated the effect of PLSCR1 on differentiation of leukemic cells involving its subcellular localization and nuclear function.

In view of the findings that activated IP3R1 stimulates the release of Ca^2+^ from the endoplasmic reticulum (ER),^24^ we investigated intracellular Ca^2+^ fluctuation in wogonoside-treated primary AML cells. Ca^2+^ acts as a pervasive intracellular second messenger that participates in essential biological processes, including secretion, cell proliferation, differentiation and motility.^25^ In this study, we showed that wogonoside stimulates the differentiation of primary AML cells via a mechanism involving the PLSCR1-IP3R1-Ca^2+^ pathway. Our findings further clarify the key effect of nucleus PLSCR1 on leukemia differentiation therapy and suggest the mechanism of wogonoside’s anti-leukemic activity, supporting the potential of developing wogonoside as a novel therapeutic agent for clinical treatment of AML patients.

## Results

### Wogonoside enhances the expression and nuclear distribution of PLSCR1 in primary AML cells

**Figure 1:**
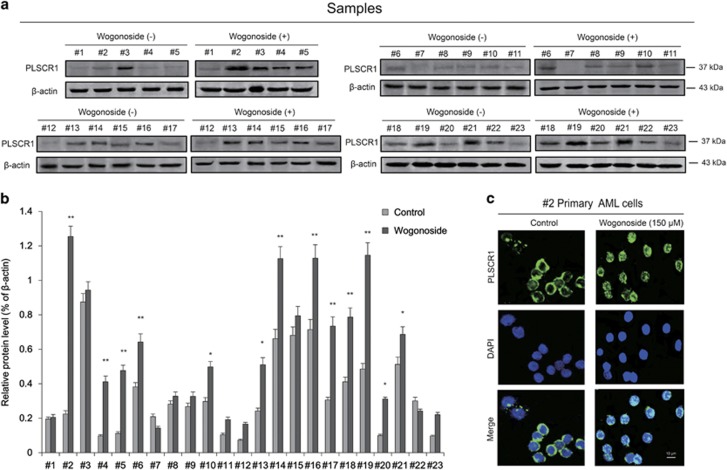
Wogonoside influences the expression and nuclear translocation of PLSCR1 in primary leukemic cells from AML patients. (**a**) Primary AML cells were incubated with or without wogonoside (150 *μ*M) for 96 h, the effect of wogonoside on PLSCR1 protein expression level was measured by western blot in primary cells from 23 clinic AML samples, *β*-actin as loading controls. (**b**) Data represent the mean±S.E.M. from three independent experiments. Asterisks denote statistically significant (*P<0.05 and **P<0.01) differences compared with controls by one-way ANOVA. (**c**) Immunofluorescence of 150 *μ*M wogonoside-treated #2 primary AML cells for 48 h costained with anti-PLSCR1 (primary)/FITC-labeled donkey anti-goat (secondary) antibody combinations (green fluorescence), as well as DAPI (blue fluorescence), to visualize the nuclei. They were detected by confocal microscopy (FV1000; Olympus) with FV10-ASW2.1 acquisition software (Olympus) at room temperature. (Original magnification × 1000; immersion objective × 100 with immersion oil type F). Images are representative of three independent experiments

To verify the effects of wogonoside on PLSCR1 expression in primary AML cells, we evaluated the PLSCR1 expression level in primary cells from 23 clinical AML patients after wogonoside (150 *μ*M) treatment (Figures 1a, b and Table 1). Results showed that samples (#1, #7, #8, #9, #11, #12, #22 and #23) with low background PLSCR1 expression and samples (#3 and #15) with high background PLSCR1 expression were not responsive to wogonoside. However, wogonoside increased the expression level of PLSCR1 in primary AML cells derived from 13 samples (the 13 samples include #2, #4, #5, #6, #10, #13, #14, #16, #17, #18, #19, #20 and #21 samples). Sample #2 whose PLSCR1 expression was most significantly increased by wogonoside was chosen to further investigate subcellular distribution of PLSCR1 in response to wogonoside (150 *μ*M) in primary AML cells (Figures 1a and b). As shown in Figure 1c, wogonoside (150 *μ*M) treatment not only increased the expression of PLSCR1 in #2 primary AML cells, but also promoted translocation of PLSCR1 into nucleus.

### Wogonoside facilitates the binding of PLSCR1 to the IP3R1 promoter and affects the expression of PLSCR1-associated cell cycle- and differentiation-related proteins

**Figure 2:**
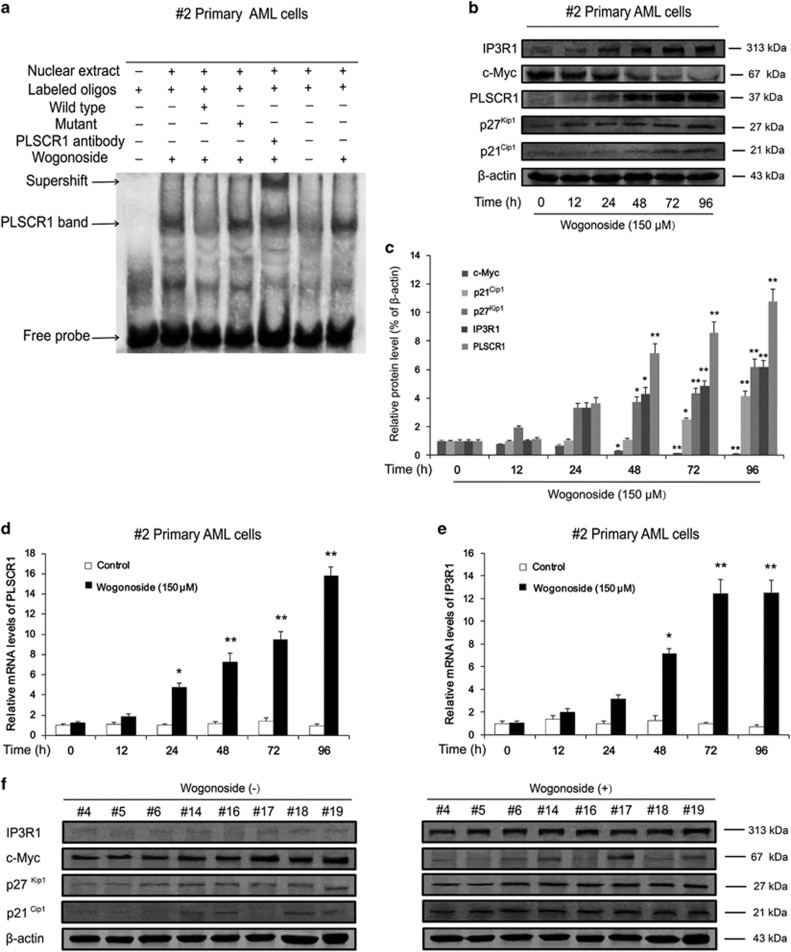
Wogonoside facilitates PLSCR binding to the IP3R1 promoter and influences the expression of cell cycle- and differentiation-related proteins and genes in primary AML cells. (**a**) Data of EMSA assay to detect PLSCR1 binding to its consensus site in the IP3R1 promoter is shown. #2 Primary AML cells were incubated with wogonoside (150 *μ*M) for 48 h, and DNA binding was determined in nuclear extracts using EMSA. To determine the composition of the DNA-binding complex, the anti-PLSCR1 antibody was used for supershift experiments. Data are representative of three separate experiments. (**b** and **c**) #2 Primary AML cells were treated with or without 150 *μ*M wogonoside for 0, 12, 24, 48, 72 and 96 h. Whole-cell extracts at different time points were analyzed by western blot for PLSCR1 and cell cycle- and differentiation-related proteins, including p21^Cip1^, p27^Kip1^, c-Myc, IP3R1, using *β*-actin as a loading control. In western blot, the amounts of cell extract in each gel were exactly equal in analysis for purpose proteins; moreover, the experiment condition and scanning parameter were permanent. The data represent the mean±S.E.M. of three different experiments. Asterisks denote statistically significant (*P<0.05 and **P<0.01) differences compared with controls by one-way ANOVA. (**d** and **e**) Total RNAs were extracted at the indicated time points. PLSCR1 and IP3R1 mRNA levels were detected by quantitative real-time reverse transcription-PCR, and fold changes were assessed and shown normalized to GAPDH (glyceraldehyde-3-phosphate dehydrogenase) mRNA level. For analysis of RT-PCR results, asterisks denote significant (*P<0.05 and **P<0.01) differences relative to controls by two-tailed Student’s tests. (**f**) Primary AML samples (#4, #5, #6, #14, #16, #17, #18 and #19) were treated with or without 150 *μ*M wogonoside for 96 h. Whole-cell extracts at different time points were analyzed by western blot for PLSCR1 and cell cycle- and differentiation-related proteins, including p21^Cip1^, p27^Kip1^, c-Myc and IP3R1, using *β*-actin as a loading control

Our previous study demonstrated that wogonoside promotes translocation of PLSCR1 into nucleus and facilitates its binding to the *IP3R1* promoter region in U937 and HL-60 cells.^7^ Similar results were observed in primary AML cells, wogonoside enhanced the DNA-binding activity of PLSCR1 to the *IP3R1* promoter region in #2 primary AML cells treated with 150 *μ*M wogonoside for 48 h (Figure 2a). Consistently, both mRNA and protein expression levels of IP3R1 were significantly increased (Figures 2b, c and e). These results confirmed that the effects of wogonoside on the translocation of PLSCR1 into nucleus and the binding activity of it to *IP3R1* promoter region in #2 primary AML cells were consistent with the AML cell lines.

Moreover, wogonoside (150 *μ*M) exerted significant effects on the expression of several cycle- and differentiation-related proteins, including upregulation of PLSCR1, p21^Cip1^ and p27^Kip1^ and downregulation of c-Myc. Results of western blot analysis of whole-cell lysates showed that the total expression of PLSCR1 was increased in #2 sample cells after treatment with 150 *μ*M wogonoside for 24 h. Following elevation of intracytoplasmic PLSCR1, expression of cyclin p27^Kip1^ was markedly increased. Furthermore, levels of p21^Cip1^ and IP3R1 were significantly upregulated and c-Myc markedly inhibited after treatment with 150 *μ*M wogonoside for 48 h (Figures 2b and c). To further investigate whether wogonoside exerts its effects on cell differentiation via modulating DNA transcription, PLSCR1 and IP3R1 mRNA levels in #2 primary AML cells were examined in the presence of wogonoside at a concentration known to induce differentiation (150 *μ*M) for 0, 12, 24, 48, 72 and 96 h. Reverse transcription-polymerase chain reaction (RT-PCR) results showed that PLSCR1 mRNA expression started to increase at 24 h, and reached a relatively high level after 48 h of wogonoside treatment. The *IP3R1* level was increased at the 48-h time point of wogonoside treatment (Figures 2d and e). Furthermore, similar to the results of sample #2, expression levels of IP3R1, p21^Cip1^ and p27^Kip1^ were all increased and c-Myc markedly inhibited after treatment of wogonoside for 96 h in another eight AML samples (#4, #5, #6, #14, #16, #17, #18 and #19) whose PLSCR1 expression levels were markedly upregulated by wogonoside (Figure 2f). Our results collectively suggest that wogonoside increased the expression of PLSCR1 and its related cell cycle and differentiation proteins and enhanced mRNA levels of PLSCR1 and IP3R1.

### PLSCR1 deficiency suppresses wogonoside-induced differentiation of primary AML cells

**Figure 3:**
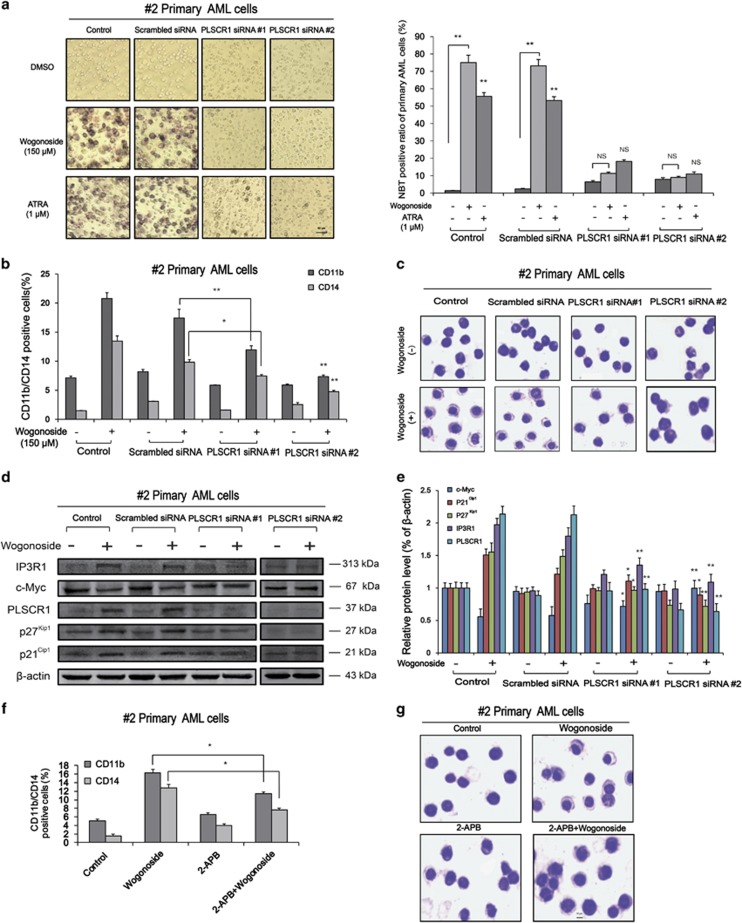
PLSCR1 and IP3R1 are involved in wogonoside-induced differentiation of primary AML cells. #2 Primary AML cells were transfected with nonspecific siRNA and PLSCR1 siRNA (#1, #2) treated with or without 150 *μ*M wogonoside or ATRA (1 *μ*M) for 96 h. Asterisks denote statistically significant (*P<0.05 and **P<0.01) differences compared with controls by one-way ANOVA. (**a**) The NBT-positive ratio of primary AML cells is shown. NBT-positive cells with purple-black color were counted, and the overall percentage was calculated based on 200 total cells per microscopic field and counting five times in each group. (**b**) CD11b and CD14 expression of primary AML cells were detected by flow cytometry analyses. CD11b- and CD14-positive ratio of primary AML cells is shown; columns represent means of three different experiments; bars represent S.E.; (**c**) Representative Wright–Giemsa staining for morphological examination is shown. Original magnification was × 400 (objective lenses × 40) under a light microscope (IX51; Olympus), and images were captured using DP2-BSW software (Olympus) at room temperature. (**d** and **e**) Confirmation of the silencing of PLSCR1 expression and the effects of silencing PLSCR1 on the expression of cell cycle- and differentiation-related proteins, which could be influenced by wogonoside, were detected by western blot with *β*-actin as a loading control. The data represent the mean±S.E.M. of three different experiments. (**f**) Confirmation the effect of inhibiting IP3R1 on the differentiation. #2 Primary AML cells were cultured for 96 h with or without 150 *μ*M wogonoside after a 1- h preincubation period with 50 *μ*M 2-APB. The percentages of cells expressing CD11b and CD14 were detected by flow cytometry analyses. The data represent the mean±S.E.M. of three different experiments. (**g**) Representative Wright–Giemsa staining for morphological examination is shown. Original magnification was × 400 (objective lenses × 40) under a light microscope (IX51; Olympus), and images were captured using DP2-BSW software (Olympus) at room temperature

**Figure 4:**
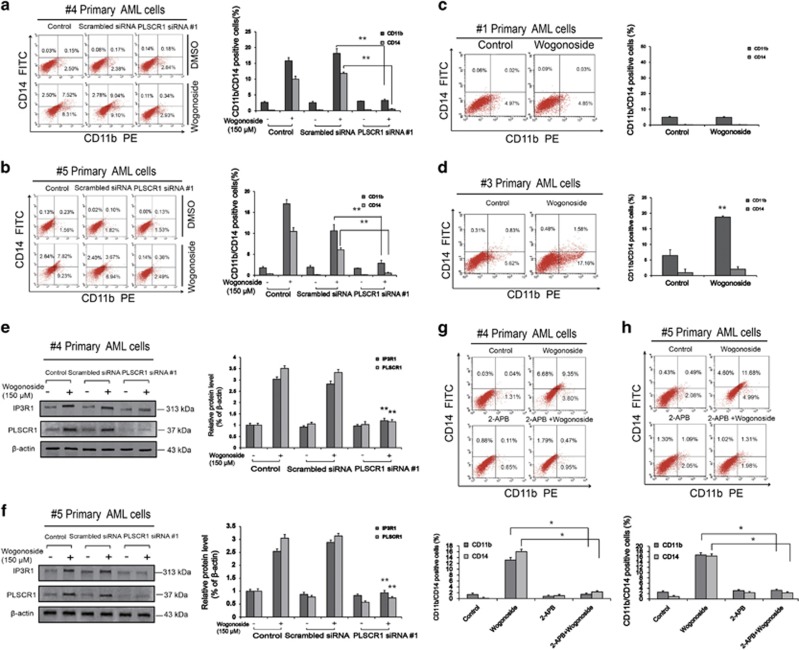
The differentiation induction effects of PLSCR1/IP3R1 on different PLSCR1-response AML samples. #4 and #5 Primary AML cells were transfected with nonspecific siRNA and PLSCR1 siRNA #1 treated with or without 150 *μ*M wogonoside for 96 h. #1 and #3 Primary AML cells were treated with or without 150 *μ*M wogonoside for 96 h. Asterisks denote statistically significant (*P<0.05 and **P<0.01) differences compared with controls by one-way ANOVA. (**a–d**) CD11b and CD14 expression of primary AML cells were detected by flow cytometry analyses. CD11b- and CD14-positive ratio of primary AML cells is shown; columns represent means of three different experiments; bars represent S.E. (**e** and **f**) Confirmation of the silencing of PLSCR1 expression and the effects of silencing PLSCR1 on the expression of IP3R1, which could be influenced by wogonoside, were detected by western blot with *β*-actin as a loading control. The data represent the mean±S.E.M. of three different experiments. (**g** and **h**) Confirmation the effect of inhibiting IP3R1 on the differentiation. Primary AML cells were cultured for 96 h with or without 150 *μ*M wogonoside after a 1-h preincubation period with 50 *μ*M 2-APB. The percentages of cells expressing CD11b and CD14 were detected by flow cytometry analyses. The data represent the mean±S.E.M. of three different experiments

To investigate whether the differentiation-promoting effect of wogonoside on primary AML cells is dependent on PLSCR1 expression, cells (samples #2 and #19) were transfected with PLSCR1 small interfering RNA (siRNA; #1 and #2) and the efficacy of transfection monitored using western blotting. Cell differentiation analyses were subsequently performed by using nitroblue tetrazolium (NBT) reduction assay, Giemsa staining and FACS assay. Notably, upon silencing of PLSCR1, wogonoside-induced differentiation effects on #2 and #19 primary AML cells were significantly reduced. For example, the nucleocytoplasmic ratio and the expression of CD11b and CD14 were essentially unchanged, and NBT reduction activity induced by wogonoside was basically abolished (Figures 3a and c and Supplementary Figures 1a and b). In primary cells from samples #4 and #5, we obtained similar results as sample #2 that PLSCR1 deficiency decreased wogonoside-induced expression of CD11b and CD14 (Figures 4a and b). Annexin V/PI staining indicated that wogonoside could not induce apoptosis of primary AML cells (#2, #4 and #5) (Supplementary Figures 4a-c). However, wogonoside-induced differentiation was not observed in non-responsive sample (#1) with low background PLSCR1 expression (Figure 4c). Furthermore, we observed that wogonoside-induced differentiation of sample (#3) with high background PLSCR1 expression although its expression level was barely affected, indicating that wogonoside-induced differentiation of primary AML cells was more likely due to nuclear import of PLSCR1 (Figure 4d). To investigate the effect of wogonoside on normal primary hematopoietic cells, we isolated and purified the CD34^+^ cells from umbilical cord blood (Supplementary Figure 5a). CD34^+^ cells were analyzed by FACS after treatment with wogonoside, and results showed that the expression of CD11b/CD14 was not changed by wogonoside compared with control (Supplementary Figure 5b). These findings suggested that PLSCR1 and its nuclear translocation have important roles in wogonoside-induced differentiation of primary AML cells.

### IP3R1 inactivation suppresses wogonoside-induced differentiation of primary AML cells

To further define the regulatory effect of PLSCR1-IP3R1 signaling activation on wogonoside-induced differentiation of primary AML cells, PLSCR1 siRNA (#1, #2) was used to analyze the expression of PLSCR1-IP3R1-related cell cycle and differentiation proteins in #2 and #19 primary AML cells. With PLSCR1 silencing, wogonoside-induced upregulation of IP3R1 was reduced (Figures 3d, e, Supplementary Figures 1c and d). Moreover, 2-APB, IP3R1 inhibitor, could decrease wogonoside-induced differentiation of #2 primary AML cells, indicating IP3R1 had important role in PLSCR1-mediated differentiation effects (Figures 3f and g). In primary cells from samples #4 and #5, we obtained similar results that IP3R1 inactivation suppresses wogonoside-induced differentiation of primary AML cells as sample #2 (Figures 4e and h). Moreover, in primary AML cells from samples #2 and #19, wogonoside exerted significant effects on several cycle- and differentiation-related proteins, including p21^Cip1^ and p27^Kip1^ upregulation and c-Myc downregulation, which could be reversed by PLSCR1 silencing (Figures 3d, e, Supplementary Figures 1c and d). Collectively, these results confirm the involvement of PLSCR1-IP3R1 signaling activation in wogonoside-induced differentiation of primary AML cells.

### Effects of wogonoside on U937 xenograft model and primary AML cell-bearing nonobese diabetic/severe combined immunodeficient (NOD/SCID) mice

**Figure 5:**
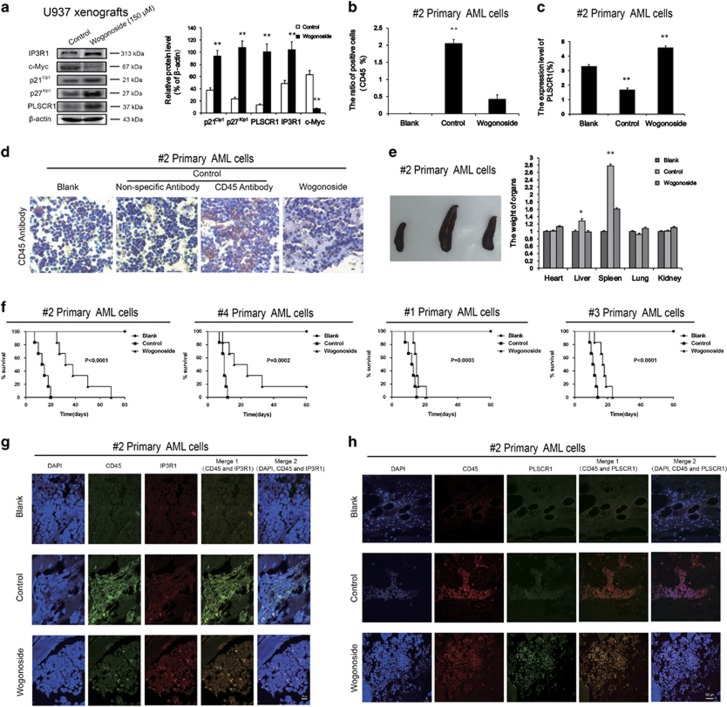
Effects of wogonoside on U937 xenografts model and primary AML cells-bearing NOD/SCID mice. U937 xenografts model BALB/c nude mice and primary AML cells-bearing NOD/SCID mice are shown. Results are representative of three independent experiments. Data represent the mean±S.E.M. of three different experiments. Asterisks denote statistically significant (**P<0.01) differences compared with controls by one-way ANOVA. Animals were observed for 80 days after cell injection. (**a**) Cell cycle- and differentiation-related proteins including p21^Cip1^, p27^Kip1^, PLSCR1, c-Myc, IP3R1, come from U937 xenografts mice carcinomas tissues were detected by western blot, *β*-actin was used as a loading control. (**b** and **c**) CD45 and PLSCR1 expression were examined in blood samples from three mice (#2) of each group in by flow cytometry analyses. (**d**) Histology of murine BM engrafted with #2 primary AML cells. BM samples from three mice of each group were collected and sections were performed immunohistochemistry and stained with huCD45. (**e**) Effects of wogonoside on weights of main organs in different groups (#2), and the typical photos of the spleen. Each data point represents the mean±S.D. of five animals for each group. (**f**) Kaplan–Meier survival plots for primary AML cells (#2, #4, #1, #3)-bearing NOD/SCID mice are shown. The results are representative of two separate experiments. Animals were observed for 80 days after cell injection. The survival curves differed significantly between the wogonoside-treated group and the control group. Wogonoside prolonged survival in mice compared with controls (*P*<0.001; log-rank test). (**g**) Histology of murine BM engrafted with #2 primary AML cells. BM samples from three mice of each group were collected and sections were performed Immunofluorescence and costained with huCD45-FITC (green fluorescence) and anti-IP3R1 (primary)/Alexa Fluor 594 donkey anti-mouse (secondary) antibody combinations (red fluorescence), as well as DAPI (blue fluorescence). (**h**) BM samples from three mice (#2) of each group were collected and sections were performed. Immunofluorescence and costained with huCD45-PE (red fluorescence) and anti-PLSCR1 (primary)/Alexa Fluor 488 donkey anti-goat (secondary) antibody combinations (green fluorescence), as well as DAPI (blue fluorescence). They were detected by confocal microscopy (FV1000; Olympus) with FV10-ASW2.1 acquisition software (Olympus) at room temperature (original magnification × 1000; immersion objective × 100/ × 60 with immersion oil type F). Images are representative of three independent experiments

To further investigate the effects of wogonoside on cell cycle arrest and differentiation *in vivo*, we assessed the expression patterns of cycle- and differentiation-related proteins in U937 xenografts of BALB/c nude mice.^26^ Similar to the *in vitro* results, levels of PLSCR1, p21^Cip1^, p27^Kip1^ and IP3R1, the tissue proteins of U937 xenografts, were all increased after administration of wogonoside. On the other hand, the c-Myc level was decreased with wogonoside treatment (Figure 5a).

In a NOD/SCID mouse model engrafted with primary human AML cells (sample #2) via tail vein,^27^ after administration of wogonoside for 60 days, whole blood was detected using FACS analysis. The population of human CD45^+^ leukocytes was significantly increased compared with the blank group. Notably, wogonoside administration decreased the human CD45^+^-positive leukocytes in peripheral blood of mice (Figure 5b), suggesting that wogonoside reduced the number of leukemia cells in AML-bearing NOD/SCID mice. In addition, wogonoside promoted the expression of PLSCR1 in CD45^+^-positive leukocytes of peripheral blood (Figure 5c), indicating that PLSCR1 upregulation by wogonoside in primary AML cells could be observed *in vivo*, corroborating our *in vitro* studies. Immunohistochemical (IHC) staining for CD45 revealed that engrafted AML cells had located and proliferated in BM of transplanted mice (Figure 5d). On the other hand, in wogonoside-treated group, CD45^+^ cells only slightly and sporadically distributed (Figure 5d). Subsequently, we examined the effects of wogonoside on the weights of main organs in different groups of AML-bearing NOD/SCID mice on day 60 (Figure 5e). The results showed significant increases in the weights of liver and spleen in the saline-treated control group, compared with the blank group (*P*<0.01). Wogonoside facilitated recovery and triggered tumor regression, as indicated by the low spleen weights of treated mice.^4^ The weights of other organs were not markedly changed. In addition, wogonoside noticeably prolonged survival in AML-bearing mice (samples #2, #4 and #3) compared with the control group, and slightly in mice bearing AML cells from sample #1 (Figure 5f, log-rank *P-*value<0.01). Results of hematoxylin and eosin (H&E) staining showed that samples of saline-treated control group display hepatosplenomegaly, accompanied by ballooning degeneration of liver cells and multinucleated giant cell infiltration in spleen. Lungs of mice in the saline-treated control group exhibited hyperemia of alveolar cavities, accompanied by acute inflammatory cell infiltration. In addition, saline-treated control mice had renal tubular degeneration and myocardial hypertrophy. Consistently, treatment with wogonoside relieved these symptoms to a noticeable degree in different organs (Supplementary Figure 2). Taken together, these results indicate that wogonoside promotes the expression of PLSCR1-associated cycle- and differentiation-associated proteins in U937 xenografts, and triggers tumor regression in NOD/SCID mice without detrimental effects on the status of normal organs.

We further assessed the expression of PLSCR1 and IP3R1 in CD45^+^ cells infiltrated into BM of primary AML cell-bearing NOD/SCID mice by using immunofluorescence confocal microscopy. Our results showed that wogonoside treatment noticeably increased the expression of PLSCR1 and IP3R1 and enhanced the nuclear distribution of PLSCR1 (Figures 5g and h).

### Effects of wogonoside on intracellular Ca^2+^ level in primary AML cells

**Figure 6:**
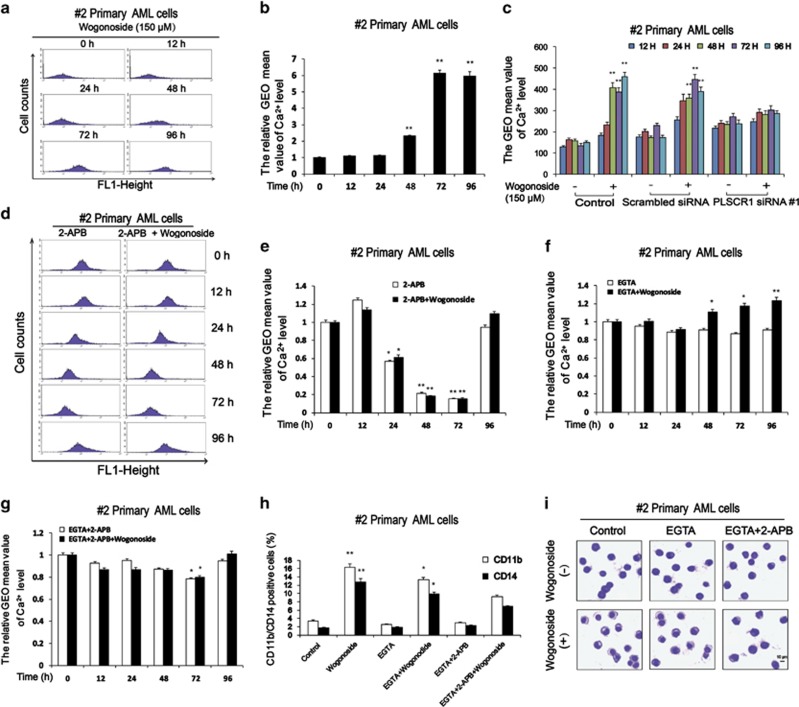
Effects of wogonoside on Ca^2+^ in primary AML cells. (**a**) Ca^2+^ level detection of #2 primary AML cells treated with or without 150 *μ*M wogonoside for 0, 12, 24, 48, 72 and 96 h was performed by flow cytometry. (**b**) The percentages of #2 cells expressing Ca^2+^ are shown. The data represent the mean±S.E.M. of three different experiments. Asterisks denote statistically significant (*P<0.05 and **P<0.01) differences compared with controls by one-way ANOVA. (**c**) #2 Primary AML cells were transfected with nonspecific siRNA and PLSCR1 siRNA #1 treated with or without 150 *μ*M wogonoside for 0, 12, 24, 48, 72 and 96 h. Ca^2+^ levels were detected by flow cytometry. Ca^2+^ level of #2 primary AML cells is shown. (**d**) #2 Primary AML cells were pretreated with 50 *μ*M 2-APB for 1 h, and then were cultured for 0, 12, 24, 48 72 and 96 h with or without 150 *μ*M wogonoside, and analyzed for Ca^2+^ expression level by flow cytometry analyses. (**e**) The percentages of #2 cells expressing Ca^2+^ are shown. (**f** and **g**) #2 Primary AML cells were pretreated with 5 mM EGTA or pretreated with 50 *μ*M 2-APB and 5 mM EGTA for 1 h, and then were cultured for 0, 12, 24, 48, 72 and 96 h with or without 150 *μ*M wogonoside, and analyzed for Ca^2+^ expression level by flow cytometry analyses. (**h**) #2 Primary AML cells were cultured for 96 h with or without 150 *μ*M wogonoside after a 1- h preincubation period with 5 mM EGTA or 50 *μ*M 2-APB and 5 mM EGTA. The percentages of cells expressing CD11b and CD14 were detected by flow cytometry analyses. (**i**) Representative Wright–Giemsa staining for morphological examination is shown. Original magnification was × 400 (objective lenses × 40) under a light microscope (IX51; Olympus), and images were captured using DP2-BSW software (Olympus) at room temperature

Our experiments validated that the differentiation effect of wogonoside results from binding of PLSCR1 to the IP3R1 promoter in primary AML cells. IP3R1 has a key role in the mobilization of intracellular Ca^2+^ stores from the ER of a variety of cells. Intracellular Ca^2+^ has been shown to be involved in an array of biological processes.^23, 25, 28, 29^ To further establish the downstream signals underlying differentiation, we examined the effect of wogonoside on intracellular Ca^2+^ level. Primary AML cells were exposed to wogonoside (150 *μ*M) for 0, 12, 24, 48, 72 and 96 h. Upregulation of intracellular Ca^2+^ level was observed at 48 h. Peak intracellular Ca^2+^ level was reached at 72 h and remained till 96 h, in the presence of 150 *μ*M wogonoside (Figures 6a and b). Silencing PLSCR1 expression by siRNA abolished wogonoside-induced upregulation of intracellular Ca^2+^ level (Figure 6c and Supplementary Figure 3). To ascertain whether cell differentiation is associated with the increased Ca^2+^ level, primary AML cells pretreated with 50 *μ*M 2-APB for 1 h, were treated with 150 *μ*M wogonoside for 12, 24, 48, 72 and 96 h. No upregulation of intracellular Ca^2+^ level was observed with a sharp decline at 48 h. However, we observed a rebound in intracellular Ca^2+^ level at 96 h (Figures 6d and e). To explore whether wogonoside-induced differentiation involves the influx of extracellular calcium, we measured intracellular Ca^2+^ level using Fluo-3AM by blocking calcium entry with ethylene glycol-bis(2-aminoethylether)-*N, N, N’, N’*-tetraacetic acid (EGTA), an extracellular Ca^2+^ chelator. In primary AML cells pretreated with EGTA, wogonoside treatment increased intracellular Ca^2+^ level compared with cells only pretreated with EGTA (Figure 6f). In order to verify the role of IP3R1 in wogonoside-induced increase of intracellular Ca^2+^, we investigated the effects of both EGTA and 2-APB. It is observed that the upregulation of intracellular Ca^2+^ level triggered by wogonoside was inhibited (Figure 6g). Moreover, upregulation of CD11b and CD14 was impaired, and wogonoside-induced differentiation was almost abrogated after incubation with 2-APB and EGTA for 96 h. However, slight changes were observed in the EGTA alone group (Figures 6h and i). These findings underscore the role of IP3R1-mediated intracellular Ca^2+^ release in wogonoside-induced leukemic cell differentiation process.

## Discussion

It has been well known that either ATRA or PMA-induced differentiation of AML cells is attributed to the upregulation of PLSCR1. However, the inducible PLSCR1 is predominantly localized outside nuclei with little distribution in the nucleus.^12^ Nuclear trafficking of newly expressed PLSCR1 has been observed only following transcriptional activation by IFN.^30^ Here, we found that PLSCR1 expression was increased by wogonoside (150 *μ*M) in primary AML cells derived from 13 samples (the 13 samples include #2, #4, #5, #6, #10, #13, #14, #16, #17, #18, #19, #20 and #21 samples) in all 23 peripheral blood samples of AML patients. In sample #2, upregulation of PLSCR1 was observed either outside or inside nuclei after wogonoside treatment, and it was also detected in peripheral blood cells of AML-bearing NOD/SCID mice. It is known that PLSCR1 is imported into the nucleus where it binds genomic DNA.^21^ Indeed, nuclear translocation of PLSCR1 was observed after treatment with wogonoside for 48 h in primary AML cells when expression of PLSCR1 was significantly upregulated, indicating that expression upregulation and nuclear translocation of PLSCR1 may be the common cause of differentiation induction. The nuclear-localized PLSCR1 specifically binds to a segment of the 5′-promoter of *IP3R1* in a nucleotide sequence-specific manner, enhancing transcription of this gene.^23^ IP3R1 is known to have a central role in IP3-mediated mobilization of intracellular Ca^2+^ stores from the ER of diverse cells and is required for cell growth, maturation and differentiation.^23, 28, 29^ In our study, IP3R1 was detected in CD45^+^ cells, which were sporadically distributed in BM of wogonoside-treated AML-bearing NOD/SCID mice, suggesting it may be associated with wogonoside’s anti-leukemia effects. Moreover, our *in vitro* study showed that wogonoside promoted the nuclear import of PLSCR1 and facilitated its binding to the *IP3R1* promoter sequence, transcriptionally activated IP3R1 expression, and triggered the release of Ca^2+^ from ER in primary AML cells. Further investigation showed that Ca^2+^, downstream of IP3R1, is significantly upregulated starting at 48 h, and reaches a peak plateau at 72 h in the presence of 150 *μ*M wogonoside. Silencing PLSCR1 by siRNA reversed the elevation of Ca^2+^ level induced by wogonoside, and Ca^2+^ level was also inhibited by 2-APB, an inhibitor of IP3R1, and underwent a sharp decline at 48 h. However, we observed a rebound in Ca^2+^ level at 96 h, suggesting the involvement of exogenous Ca^2+^. 2-APB has been reported to elicit both stimulatory and inhibitory effects on Ca^2+^ influx through CRAC channels.^31^ So EGTA was used to eliminate the influence of exogenous Ca^2+^ and involvement of CRAC channels. When extracellular Ca^2+^ was removed by EGTA, wogonoside still increased intracellular Ca^2+^ level and this effect was inhibited by 2-APB. Wogonoside-induced differentiation was almost abrogated after incubation with 2-APB for 96 h, suggesting that elevated intracellular Ca^2+^ level has a key role in the differentiation process of leukemic cells. Removing extracellular Ca^2+^ by EGTA could not eliminate the differentiation effects as 2-APB did, indicating that wogonoside-induced differentiation effects was dependent on intracellular Ca^2+^ level but not extracellular Ca^2+^ influx. Based on these findings, we speculate that the PLSCR1-pathway is responsible for wogonoside-induced primary AML cell differentiation.

Except the effect on regulation of IP3R1 and Ca^2+^, our study demonstrates that PLSCR1-related molecular events induced by wogonoside in either primary AML cells or U937 xenografts are parallel to those observed in AML cell lines. Previous studies have demonstrated that p27^Kip1^ and p21^Cip1^ are required for leukemic cell differentiation.^32, 33, 34^ Moreover, PLSCR1 induction significantly elevated p27^Kip1^ protein by inhibiting its degradation and increases p21^Cip1^ by increased transcription and reduced degradation, when c-Myc protein level can be decreased.^12^ Consistently, during wogonoside-induced myeloid differentiation of primary AML cells, significant upregulation of p21^Cip1^ and p27^Kip1^ expression was observed, and the c-Myc level was significantly decreased. PLSCR1 silencing by siRNA in primary AML cells led to almost complete abrogation of wogonoside-induced myeloid differentiation, p27^Kip1^/p21^Cip1^ upregulation and c-Myc downregulation, indicating that wogonoside-induced effects on p27^Kip1^, p21^Cip1^ and c-Myc probably are mediated by PLSCR1. Our results showed that p27^Kip1^ level started to increase when extra-nuclear PLSCR1 was upregulated (12 h), the time point earlier than either p21^Cip1^ upregulation (72 h) or c-Myc downregulation (48 h) happened, suggesting different regulatory ways. We speculate that the expression of p27^Kip1^ would be affected by cytoplasmic PLSCR1, and p21^Cip1^ and c-Myc would more likely be regulated by nuclear PLSCR1. The exact mechanism by which PLSCR1 regulates expression of these downstream proteins in wogonoside-treated primary AML cells requires further investigation.

For these experiments, primary patient-derived AML cells were selected because of their similarity to the physiological state of AML patients. The findings in our study are in good agreement with clinical observations from individuals with AML theoretically. We assessed the differentiation induction of primary cells from samples that showed different responses to wogonoside in terms of PLSCR1 expression. Wogonoside enhanced the expression of PLSCR1 and showed the highest anti-leukemia activity in samples (#2, #4 and #5) with low background PLSCR1 expression. We further observed that wogonoside-induced differentiation of sample (#3) with high background PLSCR1 expression although its expression level was barely affected. However, wogonoside-induced differentiation was not observed on non-responsive sample (#1) with low background PLSCR1 expression (Figure 4). These findings suggested that the role of PLSCR1 in AML cell differentiation is accomplished via two mechanisms: the upregulation of PLSCR1 and its nuclear translocation. The actual anti-leukemia activity of wogonoside depends on which of the two mechanisms dominates.

**Figure 7:**
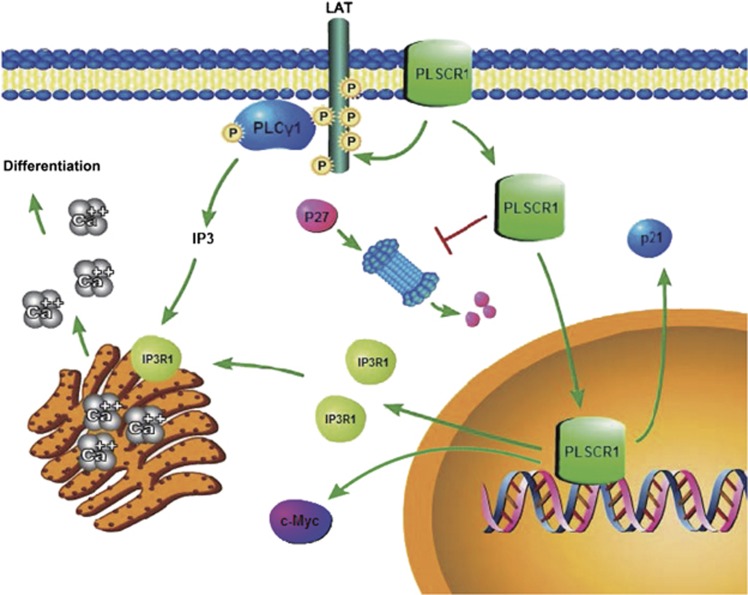
Possible mechanisms underlying the differentiation induction effect of wogonoside on primary AML cells

This study demonstrated that the nuclear PLSCR1 facilitates the PLSCR1-IP3R1-Ca^2+^ pathway leading to the differentiation of primary AML cells (Figure 7), which is responsible for wogonoside-induced anti-leukemia activity, suggesting the potential of developing of wogonoside into a novel agent for the AML treatment.

### Conclusions

Here, we investigated the contribution of wogonoside in differentiation of AML patient-derived primary cells. PLSCR1 was identified as one important protein responsible for wogonoside-induced cell differentiation through nuclear translocation. Nuclear translocated PLSCR1 facilitated its binding to *IP3R1* promoter and promoted IP3R1 expression and release of Ca^2+^ from ER, leading to differentiation of primary AML cells. Our findings provide new insight into the mechanism of wogonoside-induced differentiation and anti-leukemic effect of primary AML cells in association with activation of PLSCR1 nuclear function, which highlights a role of the PLSCR1-IP3R1-Ca^2+^ cascade, implicating the therapeutic potential of wogonoside for AML malignancies, especially for non-APL AML.

## Materials and methods

### Compounds and reagents

For *in vitro* experiments, wogonoside (98% purity; Langze Pharmaceutical Co., Ltd, Nanjing, China) was dissolved in dimethylsulfoxide (DMSO) as a stock solution at 0.5 M. Stock solution was stored at −20 °C, and freshly diluted with medium to the final concentration (150 *μ*M) before each experiment. The final DMSO concentration did not exceed 0.1%. Cells treated with the highest concentration of DMSO were used as control in the corresponding experiments. For *in vivo* analyses, wogonoside (4 mg/ml) was made into a freeze–dried power formulation by Dr. Xue Ke from College of Pharmacy, China Pharmaceutical University, and mice were injected intraperitoneally (i.p.) with or without wogonoside (80 mg/kg). ATRA was dissolved in DMSO as a stock solution at 0.01 M and used as positive control in the corresponding experiments.

NBT, EGTA and 4, 6-diamidino-2-phenylindole dihydrochloride (DAPI) were purchased from Sigma-Aldrich (St. Louis, MO, USA). 2-APB and PLSCR1 siRNA (#1) were purchased from Santa Cruz Biotechnology (Santa Cruz, CA, USA), PLSCR1 siRNA #2 was purchased from ThermoFisher Scientific (San Jose, CA, USA), and transfection was performed using Lipofectamine 2000 reagent (Invitrogen, San Diego, CA, USA), according to the manufacturer’s instructions.^35^ Fluo-3AM was purchased from Beyotime (Nanjing, China). RPMI-1640 medium and heat-inactivated fetal bovine serum (FBS) were purchased from Gibco Invitrogen Corporation (Carlsbad, CA, USA).

Fluorescein isothiocyanate (FITC) anti-human CD14 and phycoerythrin (PE) and FITC anti-human CD45 antibodies were obtained from Miltenyi Biotec Inc. (Auburn, CA, USA). PE anti-human CD11b antibodies were obtained from eBioscience (San Diego, CA, USA). Primary antibodies against p21^Cip1^, p27^Kip1^, IP3R1, c-Myc and *β*-actin were obtained from Santa Cruz Biotechnology; antibodies against PLSCR1 was obtained from Abnova (Taipei, Taiwan). IRDye 800-conjugated goat anti-mouse and goat anti-rabbit secondary antibodies were obtained from Rockland (Philadelphia, PA, USA). Alexa Fluor 488 donkey anti-goat IgG (H+L) antibody was purchased from Life Technologies (Carlsbad, CA, USA).

### Cell culture

Primary leukemic cells from newly diagnosed AML patients without prior therapy (The First Affiliated Hospital of Nanjing Medical University, Nanjing, China) were collected using lymphocyte–monocyte separation medium (Jingmei, Nanjing, China). The protocol of collection of cells from patients complied with guidelines in the Declaration of Helsinki, and was approved by the First Affiliated Hospital of Nanjing Medical University’s institutional review board and the appropriate ethics committees. A signed informed consent was obtained from each patient. Primary leukemia cells isolation was performed as described previously in Hussong *et al.*^36^ Primary leukemic cells were cultured in RPMI-1640 medium, supplemented with 10% FBS, 100 U/ml of benzyl penicillin and 100 *μ*g/ml of streptomycin in a humidified environment with 5% CO_2_ at 37 °C.

### Animal models

Female BALB/c nude mice (5–6 weeks old, weighing 18–22 g) (Slaccas Shanghai Laboratory Animal Co., Ltd, Shanghai, China) were used for the transplantation of U937 cells.^26^ Animals were subcutaneously injected with 2 × 10^6^ U937 cells in 0.1 ml matrigel (Becton Dickinson, Bedford, MA, USA). When tumors were already palpable (50-100 mm^3^), the mice were divided randomly into two groups (*n*=5 per group), a control group (0.9% normal saline) and a wogonoside-treated group (80 mg/kg). The treatment was carried out by i.p. injection every other day for 14 days. The dose was determined based on our preliminary studies (data not shown). At the end of the experiment, animals were killed and tumors were prepared for western blot.

Female NOD/SCID immunodeficient mice (6–9 weeks old) (Beijing HFK Bioscience Co., Ltd, Beijing, China) were sublethally irradiated (2.4 Gy), and were engrafted with primary human AML cells (2 × 10^6^ cells per mouse, *n*=6 per group) via tail vein in 24 h following the radiation treatment. Animals in the control group were injected with physiological saline to evaluate the effects of injection on survival. Seven days later, the mice were injected i.p. with or without wogonoside (80 mg/kg) every other day for 30 days.^27^ Next, the animals were inspected daily for 30 days. Finally, peripheral blood were prepared for flow cytometry after the human leukemia cells labeled with huCD45 and the BM were used to perform IHC and immunofluorescent staining. Besides, heart, liver, spleen, lung and kidney were collected for H&E staining.

Animals were maintained in an air-conditioned and pathogen-free environment (23±2 °C, 55±5% humidity) under controlled lighting (12 h light/day) and supplied with standard laboratory food and water *ad libitum* throughout the experimental period. The animal study was carried out according to the regulations of the China Food and Drug Administration (CFDA) on Animal Care.

### Differentiation assays

Cell differentiation was assessed by NBT reduction as previously reported.^37^ Three hundred cells were counted from three different fields for each data point.^38^ Cells were stained using Giemsa stain for morphologic assessment of differentiation. Fluorescence intensity of CD11b and CD14 was analyzed with a FACS Calibur flow cytometer (Becton Dickinson, San Jose, CA, USA).^39^ Data were based on the examination of 10 000 cells per sample selected randomly from 5 × 10^5^ cells.

### Western blot analysis

Preparation of whole-cell lysates was performed as described previously.^40^ Then equal amounts of extracts (50 *μ*g) were separated by 8–12% sodium dodecyl sulfate polyacrylamide gel electrophoresis and transferred onto the PVDF membranes (Millipore, Boston, MA, USA). The blots were incubated with specific antibodies overnight at 4 °C followed by IRDyeTM800-conjugated secondary antibody for 1 h at 37 °C. Detection was performed using the Odyssey Infrared Imaging System (LI-COR Inc., Lincoln, NE, USA).

### Immunofluorescence

Cells were collected onto the coverslips and fixed in ice-cold methanol for 10 min. Then, coverslips were permeabilized in 0.2% (v/v) Triton X-100 for 20 min and blocked with BSA buffer (PBS containing 3% BSA) for 1 h at room temperature. Then, cells were incubated with primary anti-PLSCR1 antibody (1:10) at 37 °C for 1 h and then 4 °C overnight, followed by incubation with Alexa Fluor 488 donkey anti-goat IgG (H+L) antibody (1:500) for 1 h at 37 °C. The coverslips were washed and counterstained with DAPI working solution (100 *μ*g/ml) for 20 min at room temperature. The coverslips were inverted onto slides and immersed in a mounting medium. The images were captured with a confocal microscope at × 1000 magnification (FV1000; Olympus, Tokyo, Japan).

The BM slides were permeabilized in 0.2% (v/v) Triton X-100 for 20 min and blocked with BSA buffer (PBS containing 3% BSA) for 1 h at room temperature. Then, the BM slides were incubated with primary anti-IP3R1 antibody (1:50) or anti-PLSCR1 antibody (1:10) at 37 °C for 1 h and then 4 °C overnight, followed by incubation with Alexa Fluor 594 donkey anti-mouse IgG (H+L) antibody (1:500) or Alexa Fluor 488 donkey anti-goat IgG (H+L) antibody (1:500) for 1 h at 37 °C. Next, the BM slides were incubated with CD45-FITC or CD45-PE antibody (1:10) at 37 °C for 1 h. Then slides were washed and counterstained with DAPI working solution (100 *μ*g/ml) for 20 min at room temperature. The slides were covered by coverslips and immersed in a mounting medium. The images were captured with a confocal microscope at × 1000 magnification (FV1000; Olympus).

### Electrophoretic mobility shift assay (EMSA)

EMSA assay was performed according to the modified method as described previously.^40^ A double-stranded mutated oligonucleotide, which positive-sense strand was (5′-CTTAAAGTGCAGGAGCTCTGTGGATGTGCTGCT-3′), was used to evaluate the specificity of PLSCR1-binding site in the *IP3R1* gene promoter region (5′-CTTAAAGTGCAGTAACCATGTGGATGTGCTGCT-3′), together with the complementary oligonucleotide (5′-AGCAGCACATCCACATGGTTACTGCACTTTAAG-3′) double-stranded probes. The anti-PLSCR1 antibody was used for supershift experiments. The results were photographed using a Bio-Rad phosphorimager and analyzed with Image Lab Software, Version 3.0 (Bio-Rad, Hercules, CA, USA).

### Quantitative real-time RT-PCR

RT-PCR was performed according to the manufacturer’s instructions.^41^ The primer sequences were as follows:

human PLSCR1-sense (5′-CTGACTTCTGAGAAGGTTGC-3′);

human PLSCR1-antisense (5′-GAATGCTGTCGGTGGATACTG-3′);

human IP3R1-sense (5′-TGACGAGAACCTGCCCTAT-3′);

human IP3R1-antisense (5′-TCCTTTCGCCATCTTGCT-3′);

human GAPDH-sense (5′-TCGTGGAAGGACTCATGACC-3′);

human GAPDH-antisense (5′-TCCACCACCCTGTTGCTGTA-3′).

### Transient transfection with siRNA

Cells were plated in six-well plates with fresh RPMI-1640 medium. The siRNA transfection was performed using Lipofectamine 2000 reagent according to the manufacturer’s instructions.^35^

### Detection of intracellular calcium level

Cells were collected and loaded with 1 *μ*M Fluo-3AM, which combined with Ca^2+^ and produced strong fluorescence. After incubating for 60 min at 37 °C in the dark, the cells were resuspended with 500 *μ*l PBS and the fluorescence intensity were measured by FACS Calibur flow cytometry (Becton Dickinson, San Jose, CA, USA) at Ex./Em. –488/525 nm.

### FACS analysis of whole blood

Whole blood of NOD/SCID mice killed via eyeball extirpation was depleted of red blood cells using red blood cell lysis buffer (eBioscience). For FACS analysis, cells were stained with anti-human CD45 antibodies at 4 °C for 30 min. Centrifugation at 350 *g* for 5 min at 4 °C was performed to collect total cells, followed by resuspension in 500 *μ*l PBS and detection using FACS Calibur flow cytometry. In this model, CD45^+^ cells appeared to be human leukemia cells.

To detect PLSCR1 expression, NOD/SCID mouse blood cells were stained with CD45 antibodies at 4 °C for 1 h and washed with PBS containing 0.5% BSA. After incubation with 1 × fixation/permeabilization buffer (eBioscience) for 30 min and 1 × permeabilization buffer (eBioscience) washes, cells were incubated with PLSCR1 antibody at 4 °C for 1 h and washed three times with PBS/BSA (0.1% BSA), followed by incubation with donkey anti-goat (FITC) secondary antibody at 4 °C for 1 h in the dark. Experiments were performed on an FACS Calibur flow cytometer.

### Histological analysis and IHC staining

To characterize the histological alterations, organs and BM from six NOD/SCID mice in each experimental group were immersed in 10% formaldehyde (pH 7.4) fixative for 24 h, embedded in paraffin, cut into sections 4 mm thick, and stained with H&E using standard histological techniques. IHC against CD45 was performed with standard techniques.

### Statistical analysis

All data were expressed as mean±S.D. from at least three independent experiments performed in a parallel manner. Statistical analysis of multiple group comparisons was performed by one-way analysis of variance (ANOVA) followed by the Bonferroni *post-hoc* test. Comparisons between two groups were analyzed using two-tailed Student's *t*-tests. A *P*-value <0.05 was considered statistically significant.

## Figures and Tables

**Table 1:** Clinical data for patient samples with AML

**Patient no.**	**Diagnosis**	**Source**	**PB-blast%**	**BM-blast%**	**WBC**	**FAB**	**Cytogenetics**	**Status**
1	AML	PB	92			M1	OD	New
2	AML	PB			331.08	M1	CD34-ANLL	New
3	AML	PB	82	4.8	81.5	M2b	FLT-ITD	New
4	AML	PB	88		29.5	M1	CEBPA mutation; BCR-ABL(9;22)(+)	New
5	AML	PB		87.5	43.36	M5a	CBF*β*/MYH11(+);BCR/ABL(-)	New
6	AML	PB			219.79	M1	BCR-ABL(9;22)(+), Ph (+)	New
7	AML	PB	52		4.64	M1	OD	New
8	AML	PB	92		53.48	M1	46XX[20] CD34-ANLL	Relapsed
9	AML	PB	98		156.9	M1	OD	New
10	AML	PB	96		143	M1	BCR/ABL	New
11	AML	PB			12.68	M2	OD	New
12	AML	PB			229	M1	OD	New
13	AML	PB	55	15.3	14.3	M1	OD	Relapsed
14	AML	PB	96		102.5	M1	OD	New
15	AML	PB			31.0	M1	OD	New
16	AML	PB	63.5		181.2	M0	TEL/AML1(+);C-Kit, NPM1, CEBPA, FLT3/ITD(-)	New
17	AML	PB			3.88	M3	PML-RARa(+)	Relapsed
18	AML	PB		>20	1.94	M6	OD	New
19	AML	PB	8		19.1	M5b	WT1(+)	New
20	AML	PB		<10	14.7	M5	FISH-t (15;17) PML-RARa (bcr3)	New
21	AML	PB	28		51.2	M2a	t(9;22) Bcr/abl (+); JAK2V617F(+) MLL/EVI1; MLL+	New
22	AML	PB		42	116.3	M4a	WT1 (+); FLT3-ILD (+)	Relapsed
23	AML	PB		24.5	77.4	M2b	FISH-AML/ETO (-); FLT3-ILD (+)	New

Abbreviations: BM, bone marrow; FAB, French–American–British; OD, outside diagnosis; PB, peripheral blood; Ph, pheresis; WBC, white blood cells count
