# Supplementary figures and images for: PLSCR1/IP3R1/Ca2+ axis contributes to differentiation of primary AML cells induced by wogonoside
Source: Cell Death Dis. 2017 May 11;8(5):e2768–. doi: 10.1038/cddis.2017.175 (PMC5520700; doi:10.1038/cddis.2017.175)

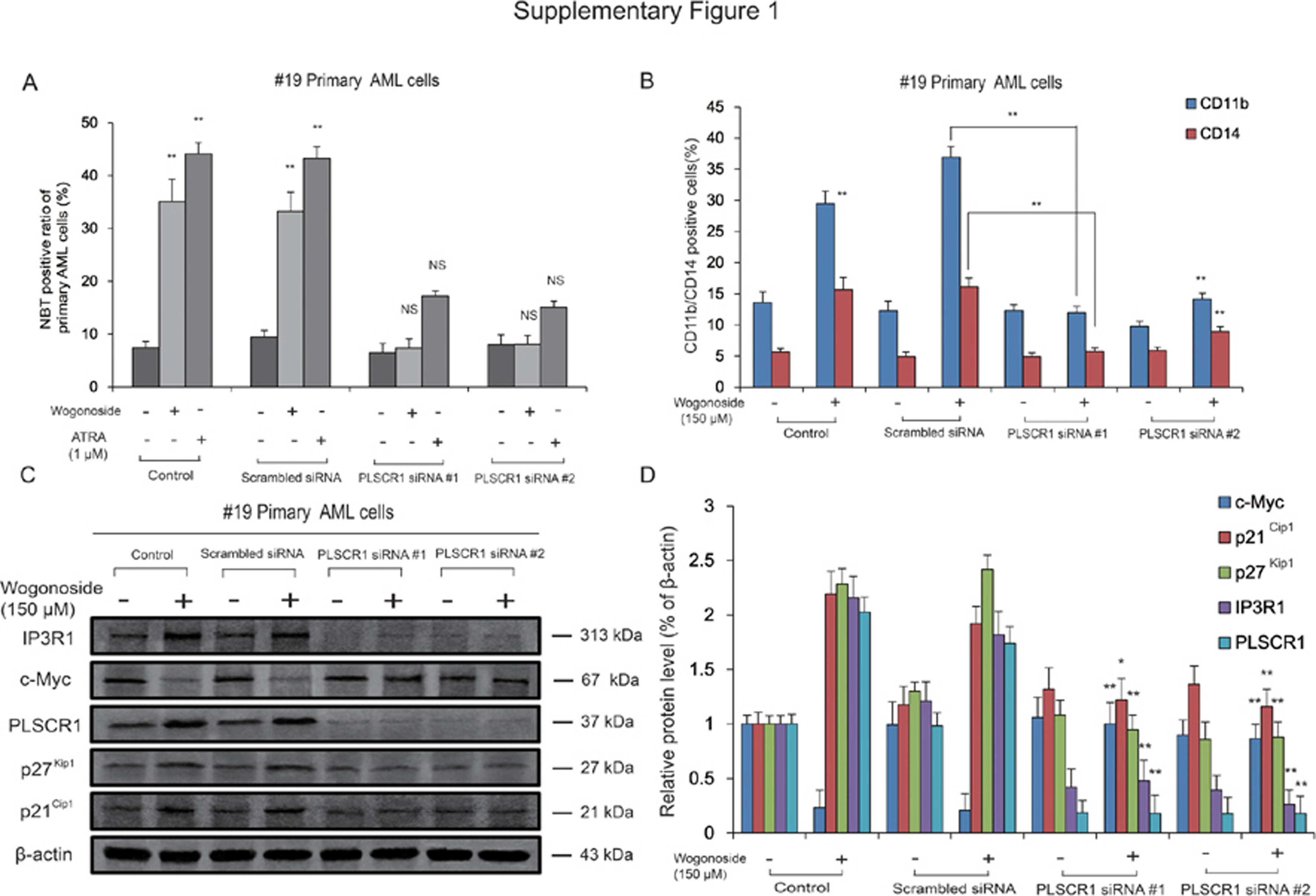

Supplement: Supplementary Figure 1 [file cddis2017175x1.tif]
