# Supplementary material for: PLSCR1/IP3R1/Ca2+ axis contributes to differentiation of primary AML cells induced by wogonoside
Source: Cell Death Dis. 2017 May 11;8(5):e2768–. doi: 10.1038/cddis.2017.175 (PMC5520700; doi:10.1038/cddis.2017.175)
Supplement: Supplementary Figure Legends [file cddis2017175x6.docx]

**Supplementary Figure Legends**

**Supplementary Figure 1:** **PLSCR1 is involved in wogonoside-induced differentiation of primary AML cells.** #19 Primary AML cells were transfected with nonspecific siRNA and PLSCR1 siRNA (#1, #2) treated with or without 150 μM wogonoside for 96 hours. Asterisks denote statistically significant (**P*< 0.05 and ***P*< 0.01) differences compared with controls by one-way ANOVA. (A) The NBT-positive ratio of primary AML cells is shown. NBT-positive cells with purple-black color were counted, and the overall percentage was calculated based on 200 total cells per microscopic field and counting 5 times in each group. (B) CD11b and CD14 expression of primary AML cells were detected by flow cytometry analyses. CD11b- and CD14-positive ratio of primary AML cells is shown; columns represent means of 3 different experiments; bars represent standard errors; (C, D) Confirmation of the silencing of PLSCR1 expression and the effects of silencing PLSCR1 on the expression of cell cycle- and differentiation-related proteins, which could be influenced by wogonoside, were detected by western blot with β-actin as a loading control. The data represent the mean ± SEM of 3 different experiments.

**Supplementary Figure 2:** H&E stains of serial sections of main organs in #2 primary AML cells-bearing NOD/SCID mice.

**Supplementary Figure 3:** #2 Primary AML cells were transfected with nonspecific siRNA and PLSCR1 siRNA #1 treated with or without 150 μM wogonoside for 0, 12, 24, 48, 72, and 96 hours. Ca^2+^ levels were detected by flow cytometry analyses.

**Supplementary Figure 4:** **The effects of wogonoside on cell apoptosis.** (A, B, C) #2, #4 and #5 Primary AML cells were treated with or without 150 μM wogonoside for 96 h to examine the apoptosis degree by Annexin V/PI double staining assay (mean ± SEM, n=3, *P< 0.05, **P< 0.01 vs. control).

**Supplementary Figure 5: Effects of wogonoside on CD34^+^ cells from umbilical cord blood.** (A) Separation efficiency of CD34^+^ cells from umbilical cord blood by human CD34 MicroBead Kit were detected by flow cytometry analyses. (B) Differention effects of wogonoside on CD34^+^ cells. The expression of CD11b and CD14 was detected by flow cytometry analyses.
